# Molecular barcoding confirms the presence of exotic Asian seaweeds (*Pachymeniopsis gargiuli* and *Grateloupia turuturu*) in the Cantabrian Sea, Bay of Biscay

**DOI:** 10.7717/peerj.3116

**Published:** 2017-03-28

**Authors:** Marcos Montes, Jose M. Rico, Eva García-Vazquez, Yaisel J. Borrell Pichs

**Affiliations:** 1 Biología Funcional, University of Oviedo, Oviedo, Asturias, Spain; 2 Biología de Organismos y Sistemas (BOS), University of Oviedo, Oviedo, Asturias, Spain

**Keywords:** Exotics, *rbcl*, COI, Seaweeds, Bay of Biscay, Rhodophyta, Introduced species, *Grateloupia*, Halymeniaceae, DNA barcoding

## Abstract

**Background:**

The introduction of exotic species can have serious consequences for marine ecosystems. On the shores of the Cantabrian Sea (North of Spain) there are no routine examinations of seaweeds that combine molecular and morphological methods for early detection of exotic species making it difficult to assess in the early stages their establishment and expansion processes as a result of anthropogenic activities (e.g., shipping and/or aquaculture).

**Methods:**

In this work we used both morphological identification and molecular barcoding (COI-5P and *rbc*L genes) of red algae collected in Asturias, Bay of Biscay (Gijón and Candás harbours) and from the University of Oviedo’s herbarium samples.

**Results:**

The results confirmed the presence of exotic Asian seaweeds *Pachymeniopsis gargiuli* and *Grateloupia turuturu* Yamada on Cantabrian Sea shores. Several individuals of these species were fertile and developing cystocarps when collected, underlining the risk of possible expansion or continued establishment. This study constitutes the first report of the Asian *P. gargiuli* in this area of the Bay of Biscay.

**Conclusions:**

Here the presence of the exotic species of the Halymeniales *P. gargiuli* is confirmed. We hypothesize that this species may have been established some time ago as a cryptic introduction with *G. turuturu* in Galician shores. The detection of these species on the shores of the Cantabrian Sea is relevant since introductions of *Pachymeniopsis* species could have been overlooked on other European coasts, probably mixed with *G. turuturu* and *P. lanceolata*. Our results confirm one new alien seaweed species that has been detected using molecular methods (COI-5P region and *rbc*L genes barcoding) on North Atlantic shores: the Asian native *P. gargiuli*. This demonstrates that routine screening for early detection of exotic algae in the Cantabrian Sea can be used for risk assessment. Genetic barcoding should be done using both *rbc*L gene and COI-5P regions since, although COI-databases are still poorer in sequences and this inhibits successful outcomes in *Grateloupia*-related species identifications, it is nonetheless a useful marker for species-level identifications in seaweeds.

## Introduction

The problem of invasion is considered one of the main threats to global biodiversity (Nunes et al., 2014). Seaweed species introductions are a significant component of Non-Indigenous Marine Species (NIMS) introductions. When these become invasive they can rapidly spread and monopolize space, alter food webs, and modify both ecosystem structure and function (Thresher, 2000; Katsanevakis et al., 2014). Shipping has been reported as the most important pathway for the introduction of NIMS and in recent years more than a thousand marine alien species have been reported in European seas, even though efforts to monitor and report alien species vary among European countries (Katsanevakis et al., 2013).

*Grateloupia* C. Agardh is the largest, least characterized and most taxonomically fluctuating genus of the family Halymeniaceae, with many poorly characterized species that display a wide array of morphological traits ranging from pinnate to subdichotomous to foliose morphologies (Guiry & Guiry, 2015; Kim et al., 2014). Recent studies (Gargiulo, Morabito & Manghisi, 2013) have resulted in a continued taxonomical rearrangement of this genus, including reinstatement of the genera *Pachymeniopsis* Y. Yamada *ex* S. Kawabata (Kawaguchi, 1997), *Prionitis* J. Agardh (Wang et al., 2001), *Dermocorynus* H. Crouan et P. Crouan (Wilkes, Mcivor & Guiry, 2005) and *Phyllymenia* J. Agardh (De Clerck et al., 2005b). Some *Grateloupia* species have also been shown to be highly invasive via marine transportation and/or aquaculture activities (e.g., Gavio & Fredericq, 2002; Nyberg & Wallentinus, 2005; Verlaque et al., 2005; Verlaque, Boudouresque & Mineur, 2007; Hewitt, Campbell & Schaffelke, 2007; Saunders & Withall, 2006; Mathieson et al., 2008; Miller, Hughey & Gabrielson, 2009; DePriest & Lopez-Bautista, 2012). *Grateloupia* is represented in the North European Atlantic by more than six species (Gavio & Fredericq, 2002; Wilkes, Mcivor & Guiry, 2005) of which only two, *G. filicina* (J.V. Lamouroux) C. Agardh, and *G. turuturu* Yamada, are considered introduced (De Clerck et al., 2005a; De Clerck et al., 2005b).

Morphological analysis alone can be ineffective for species identification and leave cryptic introductions undetected (e.g., Saunders, 2009). Molecular methods have been proven to be more effective, but morphological analysis is still the most frequent (and sometimes the only) method used for seaweed NIMS routine screenings in northern Spain (e.g., Bárbara & Cremades, 2004; Cires & Moliner, 2010). Recently four non-foliose *Grateloupia*-like samples from Gijón marina and University of Oviedo FCO Herbarium samples were identified as *G. imbricata* Holmes and the Mediterranean *G. filicina* (J.V. Lamuroux) C Agardh, two Halymeniales exotic in that area (Montes et al., 2016). This demonstrated the potential for molecular methods to detect previously overlooked species introductions and in particular, other possible introduction events for *Grateloupia* and other Halymeniales similar to the ones found on Galician coasts (*G. doryphora* and *G. turuturu*), in the coasts of the Bay of Biscay.

*Grateloupia turuturu*, a species considered highly invasive on a global scale (Nyberg & Wallentinus, 2005 as *G. doryphora* [Montagne] M.Howe) is noted as being among the invasive *Grateloupia* species on the northern coast of Spain, in Galicia (Bárbara et al., 2005; Boletín Oficial Del Estado, España, 2013). *Grateloupia turuturu* was discovered in samples considered to be *G. doryphora* that were screened using molecular identification tools (Bárbara & Cremades, 2004). Curiously, the most recent checklist for benthic algae in Asturias (Cires & Moliner, 2010) does not cite records of *G. turuturu* on Asturian coasts but it does mention the high probability of an undetected establishment. Since some confusion exists in *Grateloupia* species identification (e.g.,  Gavio & Fredericq, 2002; Kim et al., 2013; Montes et al., 2016), and since there are no routine NIMS screenings combining both molecular and anatomical methods on the coasts of the Cantabrian Sea, it is likely that a foliose *Grateloupia* species similar to the *G. doryphora* group of poorly identified species (e.g., *G. turuturu*) could be expanding via anthropogenic activities (i.e., shipping and aquaculture) as been demonstrated for many other marine species in this region (Arias et al., 2014a; Arias et al., 2014b; Habtemariam et al., 2015; Semeraro et al., 2016).

This work describes a routine screening of foliose, putatively identified *Grateloupia* specimens using morphological identification as well as COI-5P and *rbc*L genes as barcodes. Both genes were proven to be effective for species identification in red algae after blasting new sequences against BOLD (Barcode of Life Database) and GenBank databases (e.g., Saunders, 2005; Saunders & McDevit, 2012). The aim of this study was to evaluate the status of possible exotic Halymeniales introductions to the Cantabrian Sea (as outlined in Cires & Moliner, 2010).

## Materials and Methods

**Figure 1:**
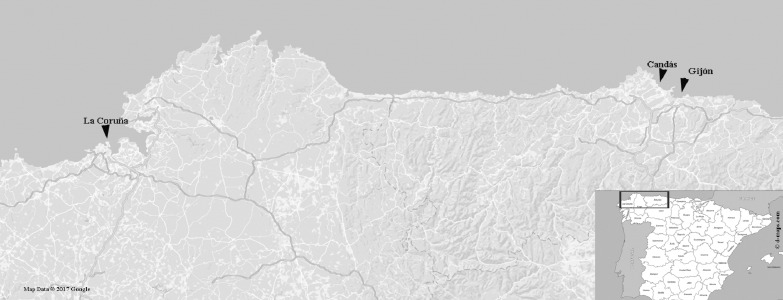
Map showing the sampling regions. La Coruña, Galicia (43°48′05″N–6°47′16″W), where FCO 1583 and FCO 1584 were collected; Candás, Asturias (43°58′79″N–5°75′85″W), where FCO 2076, FCO 2077, FCO 2135, FCO 2136, (FCO 2138–FCO 2140) were collected; and Gijón, Asturias  (43°32′43″N–5°39′44″W) where FCO 2134, (FCO 2141–FCO 2143) were collected. Map data © 2017 Google Instituto de Geografía Nacional, Spain map taken from URL: http://d-maps.com/carte.php?num_car=5674&lang=es.

In this work, a barcoding routine screening of foliose *Grateloupia* seaweeds was carried out in the large commercial port of Gijón (González, 2012 and references therein) and Candás harbour, a nearby smaller fishing and recreational harbour. Seaweed specimens were collected from jetties in the sport wharf of the inner part of the harbour of Gijón (43°32′43″N–5°39′44″W) and from the main wharf of Candás (43°58′79″N–5°75′85″W) during a low tide (Fig. 1). Samples collected were foliose Halymeniales (24 samples) preliminarily identified as *Grateloupia turuturu* on the basis of external morphology: long foliose fronds and pseudo-dichotomously branched blades of a dark reddish brown colour and mucilaginous-but-firm texture. Samples were air-dried and stored at −4 °C in the FCO Herbarium of the University of Oviedo (http://www.unioviedo.es/bos/Herbario/FCO.htm) (Table 1).

Samples already deposited at FCO were also used as external controls to this study and for species confirmation using genetic tools. The samples with voucher codes FCO 1583 and FCO 1584 were collected in 2001 from La Coruña (43°48′05″N–6°47′16″W) (Fig. 1) and classified, through classical taxonomy, as *G. doryphora*. In addition, we included the samples with the voucher codes FCO 2076 and FCO 2077, collected in 2010 from Candás (43°58′79″N–5°75′85″W) with previous morphological classifications as *G. turuturu*. Old voucher samples were rehydrated during one day in seawater prior to morphological analysis.

**Table 1:** Species assignments of the Grateloupia related algae found in Gijón, Candás and University of Oviedo Herbarium historical samples (Asturias, Bay of Biscay), using genetic data and BLAST procedures.

Location		Collector/ Date	FCO Numbers	COI-5P			*rbc*L		
				GenBank Number	Results of Assignments in BOLD System	Results of Assignments in GeneBank database.	GenBank Number	Results of Assignments in BOLD System	Results of Assignments in GeneBank database.
ASTURIAS	GIJÓN 43°32′43″N–5°39′44″W	JM Rico M Montes 4∕4∕2014	FCO 2134, (FCO 2141-FCO 2143)	**KP271163**	*Prionitis* sp. 3jeju ABMMC 11722-10 (99.7%)	*Grateloupia* sp. KJ648553.1 (99.9%)	**KP281326**	*Grateloupia* sp. AY651060.1 (100%)	*Grateloupia* sp. AY651060.1 (100%)
	CANDÁS 43°58′79″N - 5°75′85″W	JM Rico M Montes 4∕21∕2014	FCO 2135, FCO 2136, (FCO 2138–FCO 2140)						
		J Raboso. 5∕6∕2010	FCO 2076						
		J Raboso 5∕25∕2010	FCO 2077						
GALICIA	LA CORUÑA 43°48′05″N–6°47′16″W	JM Rico 9∕18∕2001	FCO 1584						
			FCO 1583	**KP271166**	*Grateloupia turuturu* ABMMC 1360-07 (100%)	*Grateloupia turuturu*KF475725.1 (100%)	**KP281329**	*Grateloupia turuturu*GU168561.1 (99,9%)	*Grateloupia turuturu*AB809603.1 (99,9%)

Freezing microtome sections and staining were carried out on all samples following Rico & Guiry (1997) using a cryotome (Leica, Germany, Model CM1510-1, Fabr. No. 2303/07.2000, Cat. No. 043631515) and blue aniline for staining, to conduct morphological analyses. DNA was extracted using the GeneMATRIX Plant and Fungi DNA purification Kit (EURx Cat. No. E3595, Roboklon GmbH, Berlin, Germany; GeneMATRIX purification Kit) using 20–70 mg of each sample for both FCO Herbarium and the fresh samples obtained at Candás and Gijón. Plant material was ground in a mortar using liquid nitrogen until pulverized material for DNA extraction was obtained. The extracted DNA was stored at −20 °C. PCRs were performed for both *rbc*L gene and COI-5P regions. Following Freshwater & Rueness (1994) and Gavio & Fredericq (2002), three different combinations of primers (F7-R753, F577-R1381 and F993-RrbcS) were used to obtain three overlapping fragments of the *rbc*L gene. The primer pair GazF1 (Saunders, 2005) and GazR4 (Saunders, 2008) were used for COI amplification. PCRs used these general conditions: 3 mM MgCl_2_, 1x of PCR Buffer, 0.4 mM dNTPs, 0.3 µM from both primers and 1u of Taq Polymerase, all in a 20 µl volume (including 2 µl of DNA extracts). PCR amplification profiles were 95 °C for 5 min; 5 cycles of 95 °C for 30 s, 42 °C annealing for 1 min, 72 °C extension for 1 min; followed by 35 cycles of 95 °C for 30 s, 46.5 °C annealing for 1 min, 72 °C extension for 1 min followed by 72 °C final extension for 10 min for COI-5P; and 95 °C for 5 min; 40 cycles of 95 °C for 1 min, 42 °C annealing for 1 min, 72 °C extension for 1 min 30 sec; followed by 72 °C final extension for 10 min for *rbc*L. These profiles are similar to those from Saunders & Moore (2013). The PCR products were electrophoresed in a 2% agarose gel, containing SimplySafe™ (EURx Cat. No. E4600-01) and using Promega 100 bp DNA Ladder Molecular Weight Marker (Promega Corporation 2800 Woods Hollow Road Madison, WI 53711, USA) for band sizes inspections. Bands were cut from agarose gels and were purified using the standard protocol of the EURx agarose purification kit (EURx Cat. No. E3540-02, Pryzodnikow, Gdansk, Poland) and sent to MACROGEN (Amsterdam, Netherlands) for sequencing using the standard Sanger sequencing method (Sanger & Coulson, 1975).

The new sequences were manually checked and edited using the freeware BIOEDIT (Hall, 1999). Alignments were made using CLUSTALW (Thompson, Higgins & Gibson, 1994). The different sequences found in this study were submitted to GenBank. After alignment and corrections, species identification was carried out using Blast to search BOLD and GenBank databases. Species identifications were accepted if they showed more than 98% similarity to the reference sequences available in both databases. Additional *Grateloupia* sequences obtained from GenBank were used in downstream phylogenetic analyses, and *Halymenia floresii* (Clemente) C. Agardh (KJ594956 and GQ862071) was used as an outgroup (Table S1). Cluster analysis was performed using the Neighbor-joining method in MEGA v6 software (Tamura et al., 2013), the Tamura-Nei DNA evolution model with invariable sites (TN93 + I) for COI-5P, and Tamura 3-Parameter with gamma distribution evolution model (T92 + G) for *rbc*L. Sequences were trimmed to 615 bp (located on the 216–831 bp region in the 3′ end) in order to include database derived sequences of varying lengths that were identified as the most likely DNA models (ModelTest software available inside MEGA v6). A total of 2,000 bootstrap steps were conducted for testing branch supports.

This study was approved by the Committee of Ethics of the Principado de Asturias, with the reference 100/06 for GRUPIN-2014-093.

## Results

### Morphology

#### Samples FCO 2077, FCO 2134, FCO 2140, FCO 2143

**Figure 2:**
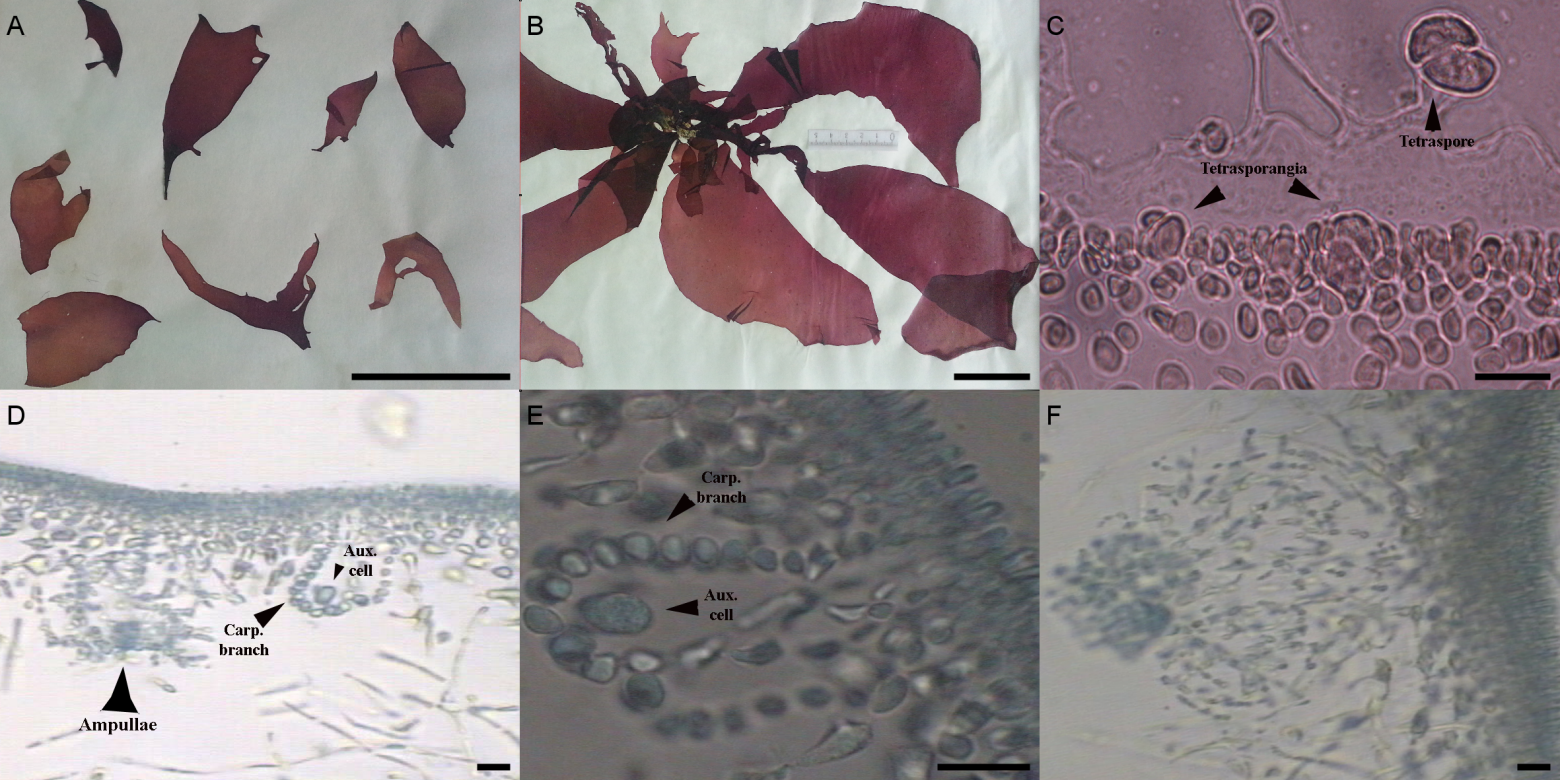
(A) *Pachymeniopsis gargiuli* from Gijón (Voucher code: FCO 2143). Scale: 5 cm. (B) *P. gargiuli* from Candás (Voucher code: FCO 2134) Scale: 5 cm. (C) Transverse section of tetrasporangia from *P. gargiuli* collected in Candas in 2010 (FCO 2077). Scale: 20 µm (D) Transversal section of *P. gargiuli* (Voucher code: FCO 2140) detailing a carpogonial branch and auxiliary cell and an “ampullae”. Scale: 20 µm (E) Transversal section of *P. gargiuli* (Voucher code: FCO 2140) detailing a carpogonial branch. Scale: 20 µm. (F) Detail of a cystocarp structure of *P. gargiuli* (Voucher code: FCO 2134). Scale: 20 µm.

The morphological characters of the University of Oviedo herbarium (FCO 2077) and freshly obtained samples (FCO 2134, FCO 2140, FCO 2143) were clearly those of the genus *Grateloupia* (Fig. 2) as they presented a habit typical of this genus, with specimens ranging from 4 to 30 cm long, with a firm texture, colors ranging from purplish-red to reddish brown and large fronds with lanceolate to linear, pseudo-dichotomously branched blades. The blades were membranaceous, lubricous and pseudo-dichotomously branched, branching from the discoid holdfast (Figs. 2A and 2B). Multiaxial section of internal structures showed a narrow cortical zone and a broad filamentous medullary zone; the cortical zone was composed of 6 layers of cells, 3 cylindrical-roundish cells of 5–10 µm long and 2 µm wide; medulla consisting of loose medullary anticlinally arranged filaments; inner cortex composed of 2–3 roundish cells of 5–10 µm diameter and extracellular mucilaginous material (Figs. 2A and 2B). The specimens found in both harbours during this study were fertile. Carpogonial branch 6-celled ampullar structure and post-fertilization events were in agreement with those in the type of the genus (Figs. 2D and 2E; Gargiulo, Morabito & Manghisi, 2013). Auxiliary cell ampullae consisted of an oval auxiliary cell, and 2–3 unbranched ampullary filaments, up to 10-cells long (Figs. 2D and 2E), similar to illustrations shown in Wilkes, Morabito & Gargiulo (2006). Tetrasporangia were detected in herbarium sample FCO 2077 and were isomorphic, arising from inner cortical cells, cruciately divided, embedded beneath the cortical surface and 25–30 µm in diameter (Fig. 2C). Cystocarps were similar to others reported both for *G. lanceolata* (Okamura) Kawaguchi (Gargiulo, Morabito & Manghisi, 2013) and to the FCO 2137 sample (Montes et al., 2016) with a diameter around 120 µm (Fig. 2F).

#### University of Oviedo herbarium sample FCO 1583

**Figure 3:**
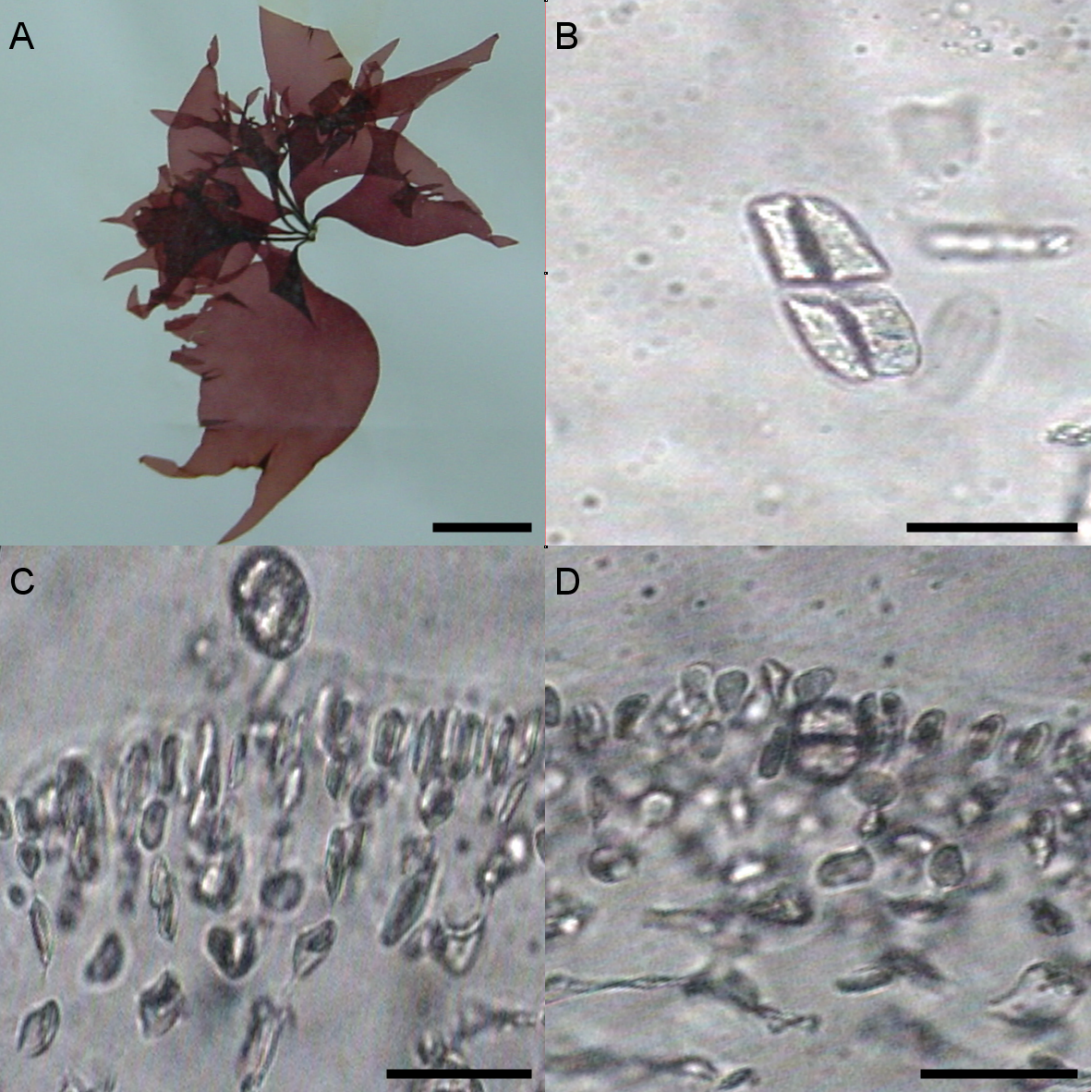
(A) *G. turuturu* from La Coruña collected in 2010 (Voucher code: FCO 1583) Scale: 3 cm. (B) Section detailing a released tetraspore. Scale: 20 µm (C, D) Transverse sections detailing images of the medullary and cortical structure of *G. turuturu* and as well tetraspores imbedded in the cortex. Scale: 20 µm.

Different morphological characteristics were found for the University of Oviedo herbarium sample FCO 1583. Fronds were 15 cm long and thinner than all the other samples (Fig. 3A). The transverse section of the middle of the frond showed a narrow cortical zone and a broad filamentous medullary zone; the cortex was formed by a 5–6 cell layer, with a conspicuous transition to the medulla (Figs. 3C, 3D). The outer cortical cells were roundish or cylindrical and 8–4 µm in diameter; medulla consisted of loose anticlinal arranged filaments. Tetrasporangia were isomorphic, arising from inner cortical cells as well but with slightly different morphology and 20–25 µm long and 5–20 µm wide (Figs. 3B–3D).

**Figure 4:**
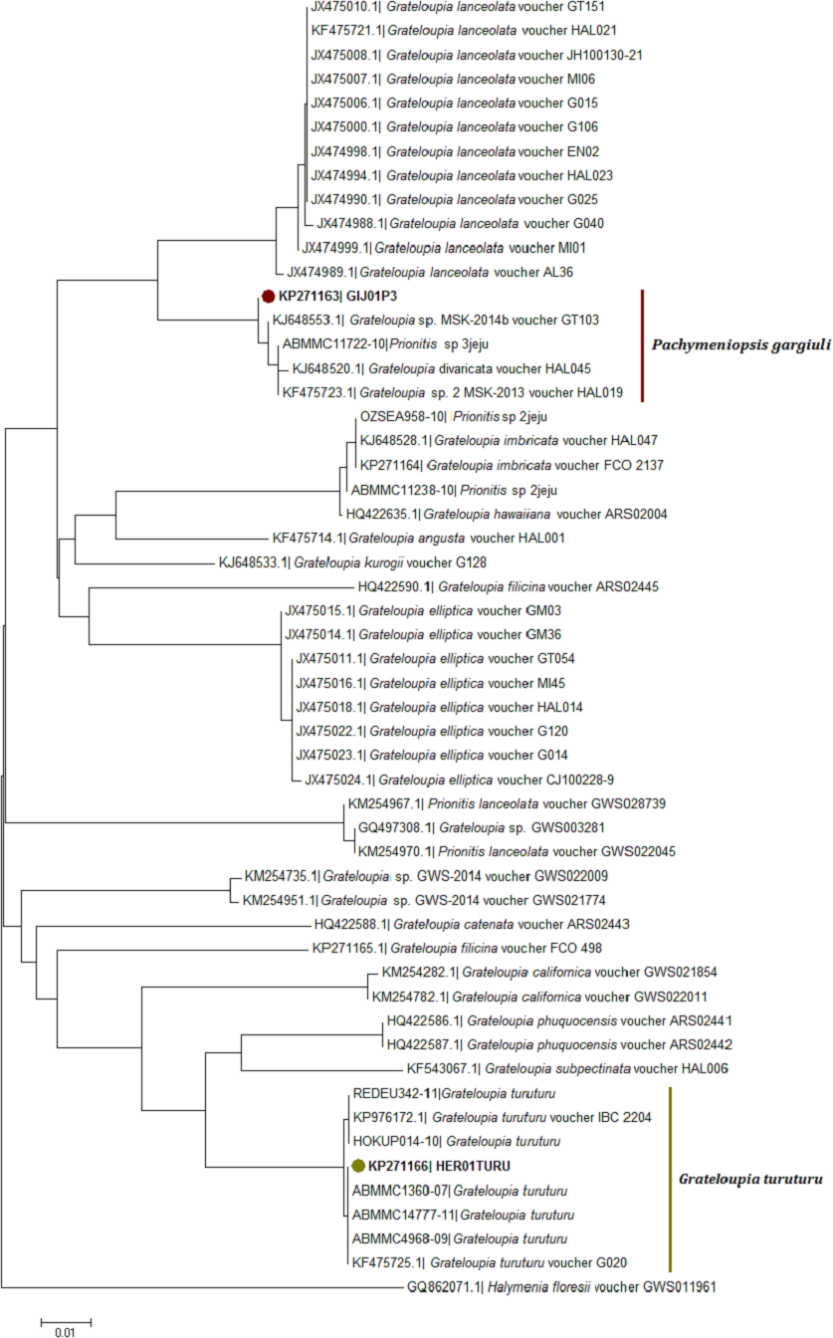
Neighbor joining consensus trees using partial sequences of COI gene. Neighbor joining consensus trees using partial sequences (530 bp) of COI gene and the DNA evolution model TN93 + I. Nodes including samples from this study appear in color.

### Genetics

Four sequences, two for each gene under analysis (*rbc*L and COI-5P), were obtained in this work. The BLAST and NJ tree analyses demonstrated that these sequences belong to two different species *Pachymeniopsis gargiuli* S.Y. Kim, A. Manghisi, M. Moribato & S.M. Boo (Kim et al., 2014) and *Grateloupia turuturu*.

#### Pachymeniopsis gargiuli

Genetic analysis of the COI-5P gene from the samples collected from Gijón and Candás (Table 1), as well as from herbarium samples FCO 2076, FCO 2077 and FCO 1584 revealed a unique COI haplotype (KP271163) of 530 bp for all of them. Analysis of the *rbc*L gene in these samples revealed also only one haplotype (KP281326) of 1,190 bp (Table 1).

Blast results for the COI KP271163 haplotype revealed unspecific genetic identification matching with an unidentified Halymeniales species found in Korea labelled as ‘*Prionitis* sp. 3jeju’ (99.7% similarity) in the BOLD database and with *Grateloupia* sp. voucher GT103 (KJ648553; 99.9% similarity) in GenBank; both vouchers came from samples collected in Asia.

Blast of the *rbc*L KP281326 haplotype against the GenBank database did not support precise genetic identification. It matched only *Grateloupia* sp. Gra017 (100% similarity), an unspecified type of *Grateloupia* found in the Straits of Messina, Italy (Wilkes, Morabito & Gargiulo, 2006).

Neighbour-joining trees were generated using all available COI-5P sequences from the two databases (Fig. 4; Table S1). The COI KP271163 haplotype was located within a very well supported branch (clade) with *Prionitis* sp. 3jeju and *G. divaricata* Okamura and an unidentified *Grateloupia* species labelled as *G.* sp. MSK 2013-14 (KJ648553, KF475723) from Korea. All these sequences formed a monophyletic clade together, apart from the highly related *G. lanceolata* (which has been synonymised with *Pachymeniopsis lanceolata* (K. Okamura) Y. Yamada ex S. Kawabata (Kim et al., 2014) (Fig. 4).

**Figure 5:**
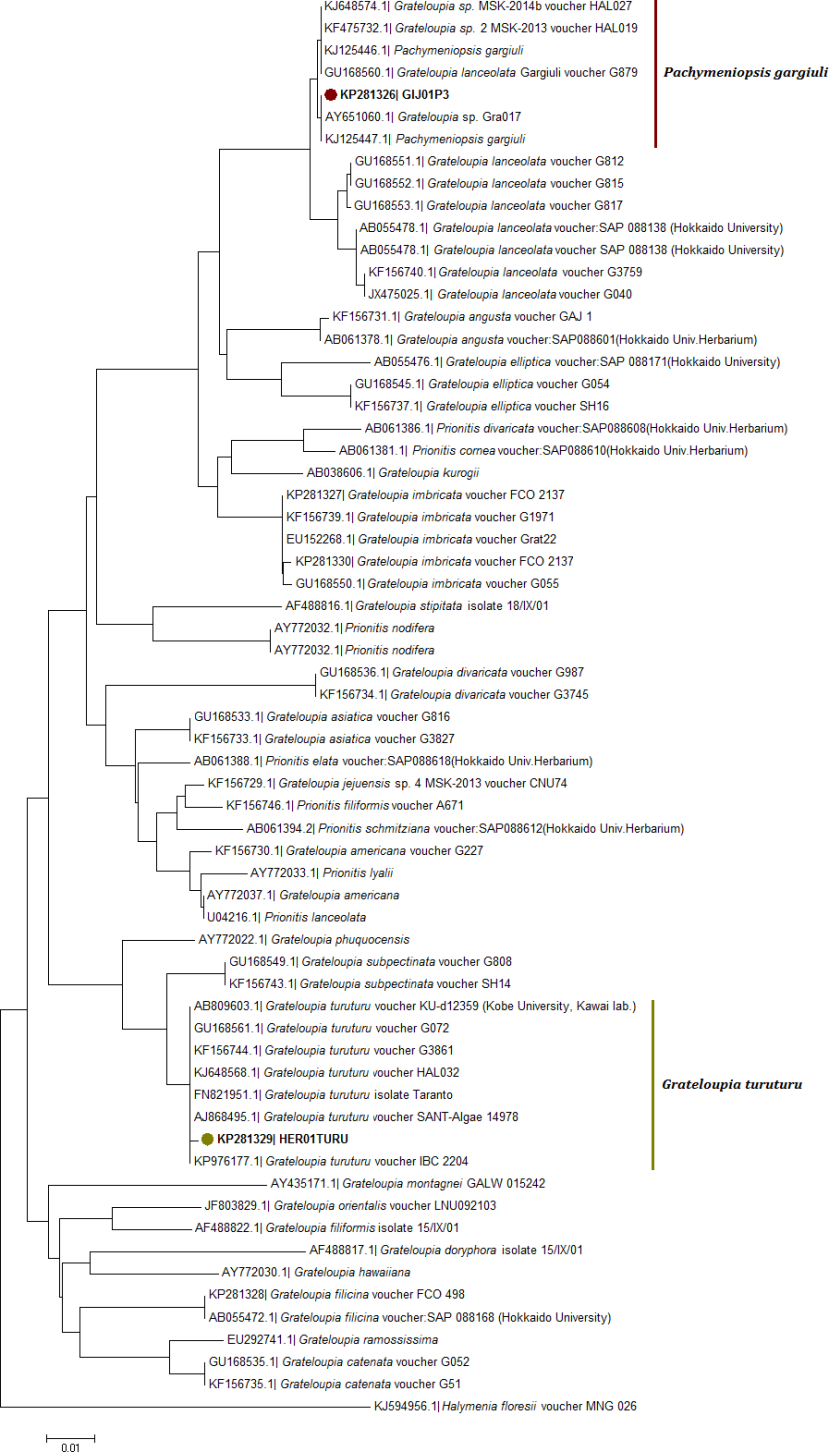
Neighbor joining consensus trees using partial sequences of *rbc*L gene. Neighbor joining consensus trees using partial sequences (615 bp, located on the 216 to 831 bp region in the 3′ end) of *rbc*L gene and the DNA evolution model T92 + G. Nodes including samples from this study appear in color.

The *rbc*L NJ tree (Fig. 5) revealed for the haplotype KP281326 a localization within a clade with KJ648574 (*G.* sp. MSK 2013) and with a species labelled as *G. lanceolata* gargiuli (GU168560) (Fig. 5). This clade has been recently identified as a gene pool grouping for the new species *Pachymeniopsis gargiuli* S.Y. Kim, A. Manghisi, M. Moribato & S.M. Boo (Fig. 5). This clade is clearly an independent entity in regard to *P. lanceolata* sequences (Fig. 5).

#### Grateloupia turuturu

Genetic analysis of the Herbarium sample FCO 1583 produced sequences for each of the genes under study, KP271166 for COI-5P and KP281329 for *rbc*L (Table 1). Blast for both genes gave a precise and specific identification. The COI KP271166 sequence showed significant similarity with *G. turuturu* using both the BOLD database and GenBank (*G. turuturu*, KF475725) (Table 1). The KP281329 sequence was also identified (99.9% similarity) as *G. turuturu* in both databases (Table 1).

The COI-5P NJ tree revealed that the sequence KP271166 was part of a well-supported monophyletic clade with other *G. turuturu* sequences from Asian areas (Fig. 4; Table S1). The *rbc*L NJ tree showed the sequence KP281329 forming a monophyletic clade with *G. turuturu* samples from various places including the native Asian areas (KJ648568 and GU168561) (Fig. 5).

## Discussion

Morphological analysis was not sufficient for precise species identification in this species complex. Despite this, we were able to determine the genus of some of the samples collected; the vegetative structure was typical of *Grateloupia*-type genera, and the reproductive structures analysis was similar to that used to separate between *Grateloupia* and *Pachymeniopsis* (Gargiulo, Morabito & Manghisi, 2013). Unfortunately not all the samples showed reproductive structures. Molecular analysis was by far the most effective method of species livel identification in this work and *rbc*L sequences supported much more precise identifications than COI sequences as previously reported for *G. imbricata* and *G. filicina* in Montes et al. (2016). This was an expected outcome given that the systematics and taxonomy of the *Grateloupia* spp. complex and related genera (e.g., *Pachymeniopsis*) have been proposed, established, discussed and rearranged using the *rbc*L gene (i.e., Wang et al., 2001; Wilkes, Mcivor & Guiry, 2005; De Clerck et al., 2005a; De Clerck et al., 2005b; Lin, Liang & Hommersand, 2008; Lee et al., 2009; Zhang et al., 2012; Gargiulo, Morabito & Manghisi, 2013; Yang & Kim, 2015). However, the COI gene has potential to become an equally effective marker for species identifications in red algae in the future as outlined in the past in other genera (Saunders, 2005; Freshwater et al., 2010; Saunders & Moore, 2013) supported by our (limited) success here. More data in COI genetic databases would help to overcome this obstacle. Moreover, in the COI-5P tree (Fig. 4) the KP271163 sequence was grouped in a complex clade including incomplete labelled species (e.g., *Grateloupia* sp. 2 MSK-2013 voucher HAL019) and one species with a correct taxonomic label, *G. divaricata* Okamura (Wang et al., 2001), a species that shows pinnated frond morphology (different from our samples), suggesting a species misidentification. This pointed to several errors in species identifications that appear in genetic databases. Fortunately, the *rbc*L tree (Fig. 5) clearly showed the correct identity since samples from this study showed 100% identity with sequences of *P. gargiuli* (KJ125446, KJ214447), a recently described species closely related to *P*. *lanceolata* as described by Kim et al. (2014).

Herbarium samples FCO 1583 and FCO 1584 were collected in the same area, the same day, and shared similar morphology; this led to both being identified as *G. doryphora* in 2001. The herbarium sample FCO 1583 has been unambiguously identified here as *G. turuturu*. However, the FCO 1584 sample was identified as *P. gargiuli*. Both samples were thus registered with incorrect species names in the FCO herbarium. *G. turuturu* presented a similar habit (Fig. 3A) to *P. gargiuli* (Figs. 2A and 2B) and vegetative and reproductive morphology was not sufficient for species identification. This underscores the value of genetic analyses if the aim is precise red algae species identifications when cryptic species complexes are considered with reproductive morphological features that are difficult to find if for example, samples do not include fertile specimens. Moreover, our results support the idea that both of the species we identified have been present in the Cantabrian Sea at least since 2001, although only *G. turuturu* has for the time being been described on Galician coasts (Bárbara et al., 2005). This last observation suggests that the presence of *P. gargiuli* in Asturias could be the result of a more recent introduction event (at least, since 2010) in comparison with other exotic seaweeds.

Cires & Moliner (2010) suggested that *G. turuturu* could be present in Asturian shores as a consequence of its proximity to coasts where it has been detected (Galicia) and because this seaweed has been reported showing invasive spread in nearby areas such as Portugal and France (Simon, Gall & Deslandes, 2001; Araújo et al., 2011), which makes expansion to other areas more probable. Although samples initially labelled by us as *G. turuturu* were collected in Asturias (including voucher samples from 2010 collected in Candás), they were found to all be *P. gargiuli*. These misidentifications are not surprising since *P. gargiuli* was also initially misidentified as *G. doryphora* and *G. turuturu* until genetic analyses were carried out on samples from the Strait of Messina (AY651060) (Wilkes, Morabito & Gargiulo, 2006). This suggests that the introduction of *P. gargiuli* in the Cantabrian Sea shores could be a cryptic introduction, thanks to its morphological similarity to *G*. *turuturu* and also to the similarity of *G. turuturu* in habit to the Galician native *G. lanceola* J. Agardh (J. Agardh). The latter similarity has resulted in a tendency to overlook the extent of *G. turuturu* presence in Galicia in the first place (Bárbara & Cremades, 2004).

*P. gargiuli* is also considered cryptic for *P. lanceolata*, sharing many morphological characteristics as well as distribution area in Korea (Kim et al., 2014). Samples of this species were detected in Italy (Wilkes, Morabito & Gargiulo, 2006) and, in the Canary Islands, initially identified as *P. lanceolata* (EU024819) (García-Jiménez et al., 2008), and also in the Madeira Islands at least since 2006; *P. lanceolata* was described, but only via morphological analysis (Ferreira et al., 2012). The similarity between these seaweeds raises the clear possibility of the presence of *P. gargiuli* in Madeira. The red algae *G. imbricata* was also found in Canarias through genetic analysis and in Madeira through classical taxonomy (García-Jiménez et al., 2008; Ferreira et al., 2012), but it has to date not been described anywhere in North Atlantic European shores or marinas except in the Gijón area (Montes et al., 2016). These similarities in locations where it was detected as an introduced species may suggest that these algae shared the same or similar introduction vectors/routes. The most likely and accepted hypothesis regarding introduction vectors of *Pachymeniopsis* spp. and of *G. turuturu* is oyster commerce to the Thau lagoon from the Pacific (Verlaque et al., 2005; Verlaque, Boudouresque & Mineur, 2007). It is likely that shipping will play a pivotal role in the range expansion of these species to the continent through ballast water and hull fouling (Hewitt, Campbell & Schaffelke, 2007).

This is the first report of a new genus, *Pachymeniopsis*, on the European shores of the North Atlantic. *P. gargiuli* is a species native to Asia, which was probably introduced long ago (at least since 2001) as a cryptic introduction within *G. turuturu* to Galician shores. Several individuals of these species were fertile as they were developing cystocarps and tetraspores when collected, emphasizing the risk of expansion or continued establishment. The detection of these species on the coasts of the Cantabrian Sea also indicates that *Pachymeniopsis* introductions may have been overlooked along other European coasts, especially if intermingled with *G. turuturu* and *P. lanceolata*. Our results highlight the potential for exotic algal introductions being missed when morphological identification fails to differentiate between highly similar species, and thus the importance of routine molecular barcoding surveys. This study also highlights the existence of gaps in the COI-5P records for *Grateloupia* spp., which might necessitate using an alternative barcode in the form of *rbc*L. We confirm previous findings and reports on two previously overlooked exotic algal introductions to an area of Europe where these had not been detected by morphology alone, the Asian native *G. imbricata* and the Mediterranean native *G. filicina* (De Clerck et al., 2005a; De Clerck et al., 2005b), and we add a newly reported species (Montes et al., 2016), also utilizing DNA barcoding, using both COI-5P region and *rbc*L gene: the exotic *P. gargiuli*. This demonstrates the utility of routine screenings that combine both anatomical investigation and barcoding procedures for early detection of exotic algae in the Cantabrian Sea. For such studies it would be ideal to combine anatomical and DNA barcoding procedures. In our case morphological examination was able to determine accurate genus placement, but for species identification, genetic methods proved to be more effective than conventional morphological identification.

##  Supplemental Information

10.7717/peerj.3116/supp-1 Supplemental Information 1 The raw sequences Montes et al Genbank sequences rbcl(2) + COI(2)

10.7717/peerj.3116/supp-2 Table S1 Additional *Grateloupia* sequences obtained from GenBank and used in downstream phylogenetic analyses

## Additional Information and Declarations

### Field Study Permissions

The following information was supplied relating to field study approvals (i.e., approving body and any reference numbers): Field experiments have been approved by the Committee of Ethics of the Principality of Asturias, with the reference 100/16 for GRUPIN-2014-093.

### DNA Deposition

The following information was supplied regarding the deposition of DNA sequences: Two different haplotypes of 530 bp were obtained in this study for the COI-5P gene. A unique haplotype (KP271163) was obtained from Gijón and Candás new samples, as well as from the herbarium samples FCO 2076, FCO 2077 and FCO 1584. Another haplotype (KP271166) was obtained from the herbarium sample FCO 1583. Two different haplotypes of 1,190 bp were obtained in this study after the analyses of the sequences of the rbcL gene in our samples (KP281326, KP281329).

### Data Availability

The following information was supplied regarding data availability: The FCO Herbarium of the University of Oviedo. FCO 2134 –FCO 2138 FCO 2139 –FCO 2143
http://www.unioviedo.es/bos/Herbario/FCO.htm.
